# The annual trend of suicide rates from 2010 to 2021 in patients with cannabis use disorder – a national registry study

**DOI:** 10.1007/s00127-024-02781-4

**Published:** 2024-10-28

**Authors:** Martin Ø. Myhre, Eline Borger Rognli, Fredrik A. Walby, Jørgen G. Bramness, Lars Mehlum

**Affiliations:** 1 Institute for Clinical Medicine, National Centre for Suicide Research and Prevention, University of Oslo, Oslo, Norway; 2 Section for Clinical Addiction Research, Division of Mental Health and Addiction, Oslo University Hospital, Oslo, Norway; 3 Department of Alcohol, Tobacco, and Drugs, Norwegian Institute of Public Health, Oslo, Norway; 4 Institute for Clinical Medicine, UiT The Arctic University of Norway, Tromsø, Norway; 5 Norwegian National Competency Centre for Drug Abuse and Mental Illness, Brumunddal, Norway

**Keywords:** Suicide, Cannabis use disorders, Alcohol use disorders, Substance use disorders, Time series

## Abstract

**Purpose:**

The temporal trend of suicide in patients with cannabis use disorder (CUD) is important to investigate, considering the recent increases in THC concentration in cannabis products. This study describes the annual suicide rates in patients with CUD from 2010 to 2021. To investigate if any change in suicide rate was specific to CUD, we compared these suicide rates with corresponding data for patients with alcohol use disorders (AUD) and other substance use disorders (SUDs).

**Method:**

The study used a time series design. We used a national registry linkage between the Norwegian Cause of Death Registry and the Norwegian Patient Registry from 2010 to 2021, including patients with CUD (ICD-10 code F12), AUD (F10), or other SUDs (F11; F13-F16; F18-F19) who died by suicide, supplemented with the total number of patients treated with specific disorders to estimate the suicide rates. The trend was analyzed by comparing the annual suicide rate to 2010 and using Poisson regression, adjusting for gender, age, and mental disorders.

**Results:**

We found increased annual incidence rate ratios for patients with CUD in 2018 (IRR = 2.14 (95% CI 1.14–3.99)) and onwards and an increasing time trend over the study period (IRR = 1.08 (1.05–1.12)). No increases in trends were found for AUD or other SUDs. The time trend for CUD was attenuated when adjusting for depressive or anxiety disorders (aIRR = 1.00 (0.92–1.08)) or other SUDs (aIRR = 0.96 (0.87–1.06)).

**Conclusions:**

Increasing suicide rates were found in patients with CUD. Comorbid anxiety and depression or other SUDs, but not other mental disorders, could partly explain these results.

**Supplementary Information:**

The online version contains supplementary material available at 10.1007/s00127-024-02781-4.

## Introduction

Suicide is a global problem accounting for a substantial number of years of life lost [1]. The suicide rate in Norway is similar to the overall suicide rate in Europe, who is lower than in many middle- and low-income countries. An important risk factor for suicide is substance use disorders [2]. Following this, it can be hypothesized that when consumption patterns or substances change this may affect the associated suicide risk.

After alcohol, cannabis is the most frequently used drug of abuse, with a mean prevalence of any use in the last 12 months in Western European countries of around 7% [3]. The prevalence of cannabis use is slightly higher in the Americas, Africa and Oceania but lower in Asia. Cannabis users have a more than 10% risk of developing an addiction, which in turn is associated with comorbid mental disorders and treatment-seeking [4]. In Norway, cannabis is by far the most used illicit drug; around one in four of those aged 16–64 years report ever having used cannabis, and use is more common in younger age groups and men [5]. A strong increase in the concentration of Δ-9 tetrahydrocannabinol (THC), the most important psychoactive component in cannabis, has been seen internationally over the past decades [6–8] and also in Norway, where analyses of drug seizures show around 30% increase in THC in hash since 2018 [9]. Also, blood samples from Norwegian apprehended drivers show increased mean concentrations of THC [10]. Parallel to the increase in THC concentrations, an increase in treatment-seeking for cannabis-related problems has been observed in Europe [11]. In Norway a similar trend is observed. The number of patients with Cannabis use disorders (CUD) treated in specialized health services increased steadily from about 2,500 patients in 2010 until a peak in of almost 4,000 patients in 2019. The number then dropped to about 3,000 patients in 2020 and 2021 [12]. Parallel to the increase in THC has the annual incidence of cannabis-induced psychosis has increased in all Scandinavian countries [13] and the population attributable fraction of cannabis use on the risk of schizophrenia has been found to increase [14]. Given this, it would be of importance to investigate other health outcomes related to cannabis.

The association between cannabis use and suicide death is inconsistent across studies [15], but it is partly explained by mental disorders [16]. A meta-analysis found that chronic cannabis use increased the risk of suicide death but that acute cannabis intoxication did not [17]. Since this meta-analysis [17], an increased risk of suicide among CUD patients has been found in the Veterans Affairs healthcare system in the US [18], a population-based cohort study from Sweden [19], and a population-based cohort study from Hong Kong [20]. In these studies, adjustment for comorbid mental disorders attenuated the risk of suicide, which is to be expected as mental disorders are strongly associated with suicide risk [21].

Alcohol use disorder (AUD) and other substance use disorders (SUDs) are also associated with an increased risk of suicide [22, 23]. We have not seen similar changes in potency or composition in these substances that parallel the development seen in cannabis. We thus opted to use these as “control-substances” in this investigation of the annual trend of suicide rates in patients with CUD.

In this study, we aimed to examine the annual suicide rate in patients with CUD, providing incidence rate ratios for single years and the overall trend up until 2021 compared to the index year 2010. Since both CUD and suicide risk are associated with mental disorders, we adjusted for comorbid mental disorders in several different models. We also included the suicide rates in patients with AUD and other SUDs to examine whether any changes could be found in broader groups of patients with SUDs or if any observed patterns were for patients with CUD exclusively.

## Methods

### Design

This study used a time series design describing the annual suicide rates between 2010 and 2021 for patients with CUD, AUD, and other SUDs.

### Data sources, cases, and population

Cases were identified through a registry linkage of all individuals who died by suicide or undetermined causes of death (X60-X84; Y10-Y34; Y870; Y872) recorded in the Norwegian Cause of Death Registry (NCDR) between January 1st, 2010 and December 31st, 2021, and who had been in contact with specialized SUD or mental health services, as recorded in the Norwegian Patient Registry (NPR) during the last year before suicide. The unique personal identifier assigned to all Norwegian citizens at birth or immigration was used to link the data. We included all individuals with any incident CUD (ICD-10 code F12), AUD (F10), or other SUDs (F11; F13-F16; F18-F19) [24] recorded in the observation period. The number of patients with SUDs who died by suicide was aggregated by year, gender, and age.

A general treatment population was identified in the NPR as the annual number of individuals with any SUD recorded (F10-F16; F18-F19) in contact with SUD or mental health services from January 1st, 2010, to December 31st, 2021. These data were aggregated by year, gender, and age. Cells with counts below three were removed when data were delivered.

The NCDR includes information on causes of death in the Norwegian population. The registry has a high degree of completeness and good data quality [25]. The NPR contains information about contacts with specialized health services in Norway. The registry contains personally identifiable information for contact with mental health services since 2008 and for SUD services from 2009. The coverage of the registry is very good from 2010 onwards [26].

### Variables of interest

For the cases, we recorded the first CUD from the NPR between 2008 and 2021. For the comparison groups, we ascertained the first diagnosis of AUD (F10) and other SUDs (F11; F13-F16; F18-F19) in patients not diagnosed with CUD. Moreover, we recorded whether the diagnosis was primary or secondary, recorded in SUD or mental health services, and the follow-up time from recording the diagnosis to suicide in person-years. We also identified any registration of comorbid mental disorders categorized using ICD-10 codes for psychosis or bipolar disorders (F20-F31), depressive or anxiety disorders (F32-F49), personality disorders (F60-F69), and ADHD (F90). In patients with CUD we also recorded if AUD or other SUDs were present. From the NCDR, we used the date of death to extract the year of the suicide, along with gender and age at the time of death. The latter was categorized into ten-year bands (10–19, 20–29, 30–39, 40–49, 50–59, 60-).

For the general treatment population, we used aggregated counts of the number of patients treated with a CUD, AUD, or other SUDs diagnosed in specialized services by year, gender, and age group.

### Statistical analysis

Differences between the groups were examined using Chi-squared tests for categorical variables and Mann-Whitney tests for continuous variables. Suicide rates were estimated from data aggregated by year, gender, and age group. We used the number of patients who died by suicide as the numerator and the general treatment population with the same diagnosis as the denominator.

We estimated overall, gender-specific, and age-specific suicide rates and the annual number of suicides and crude suicide rates from 2010 to 2021. Next, we analyzed the annual trends of suicide rates between 2010 and 2021 by estimating incidence rate ratios (IRRs), using the suicide rate in 2010 as a reference. Confidence intervals for the IRRs were estimated using the Delta method, and two-tailed Wald tests were used to test the significance of the IRRs.

To examine the trend of the suicide rates over the whole period, we used Poisson regression, regressing the suicide rate over year. We built both a crude model and a model adjusted for gender and age. Groups with missing data in the population were excluded from the gender- and age-adjusted model. Furthermore, we adjusted the crude model separately for annual counts of psychosis or bipolar disorders, depressive or anxiety disorders, personality disorders, ADHD, and AUD or other SUDs to assess whether any of these variables could explain the trends in suicide rates. In models finding a crude association between the suicide rate and year, the explanatory potential of the covariates was considered. The explanatory potential was considered present if the coefficient for the year was non-significant and the coefficient for the covariate was significant after adjustment. Data were analyzed with R version 4.2.2 [27], and the figures were generated with the ggplot2 package [28].

### Ethics and approvals

The Norwegian Directorate of Health has given an exemption from patient confidentiality (ref: 16/27835-12) to access the individual data for the cases since this is a retrospective study where informed consent is impossible to obtain. The linkage was further approved by the Regional Committee for Medical and Health Research Ethics South-East Norway (reference: 32494). The aggregated data on the population were delivered as anonymous data where no formal approvals were needed.

## Results

We identified 348 individuals registered with a CUD diagnosis who died by suicide from 2010 to 2021 and who had had contact with services for SUD or mental health within the last year before their death. In the same period, there were 565 suicides in individuals with AUD and 338 suicides in individuals with other SUDs in contact with SUD or mental health services last year before suicide. Individuals with CUD who died from suicide were more often men (78.0%) and of younger age, 75% between 20 and 39 years (34%), compared with individuals with AUD and other SUDs who died by suicide. Comorbid mental disorders were prevalent in all substance groups, especially depression or anxiety. Hanging or strangulation was a more common suicide method among patients with CUD than among patients with AUD and other SUDs (57%; *p* < 0.001). Table 1 shows other background characteristics and differences between patients with CUD, AUD, and other SUDs.

**Table 1:** Characteristics of patients with alcohol, cannabis and other substance use disorders who died by suicide in Norway in the years 2010 to 2021

Characteristic	Cannabis use disorder	Alcohol use disorder,	Other substance use disorders	*p*
*N*	348	565	338	
Gender *n* (*%*)				< 0.001^1^
Females	78 (22%)	186 (33%)	141 (42%)	
Males	270 (78%)	379 (67%)	197 (58%)	
Age *n* (*%*)				< 0.001^1^
10–19	11 (3.2%)	2 (0.4%)	4 (1.2%)	
20–29	142 (41%)	73 (13%)	62 (18%)	
30–39	117 (34%)	93 (16%)	94 (28%)	
40–49	52 (15%)	160 (28%)	74 (22%)	
50–59	15 (4.3%)	136 (24%)	66 (20%)	
60-	11 (3.2%)	101 (18%)	38 (11%)	
Suicide method *n* (*%*)				< 0.001^1^
Hanging/strangulation	199 (57%)	217 (38%)	139 (41%)	
Poisoning	67 (19%)	158 (28%)	118 (35%)	
Other methods	82 (24%)	190 (34%)	81 (24%)	
Service of diagnosis *n* (*%*)				< 0.001^1^
Mental Health Services	176 (51%)	358 (63%)	250 (74%)	
SUD services	172 (49%)	207 (37%)	88 (26%)	
SUD as primary diagnosis *n* (*%*)	145 (42%)	352 (62%)	224 (66%)	< 0.001^1^
Follow-up time *Median (IQR)*	3.9 (1.4, 7.1)	2.2 (0.7, 4.8)	2.2 (0.8, 5.2)	< 0.001^2^
Non affective psychosis or bipolar disorder *n* (*%*)	136 (39%)	145 (26%)	109 (32%)	< 0.001^1^
Depressive or anxiety disorder *n* (*%*)	231 (66%)	388 (69%)	219 (65%)	0.500^1^
Personality disorder *n* (*%*)	97 (28%)	138 (24%)	85 (25%)	0.500^1^
ADHD *n* (*%*)	63 (18%)	55 (9.7%)	39 (12%)	< 0.001^1^
Alcohol or other substance use disorders *n* (*%*)	283 (81%)	-	-	-

**Fig. 1:**
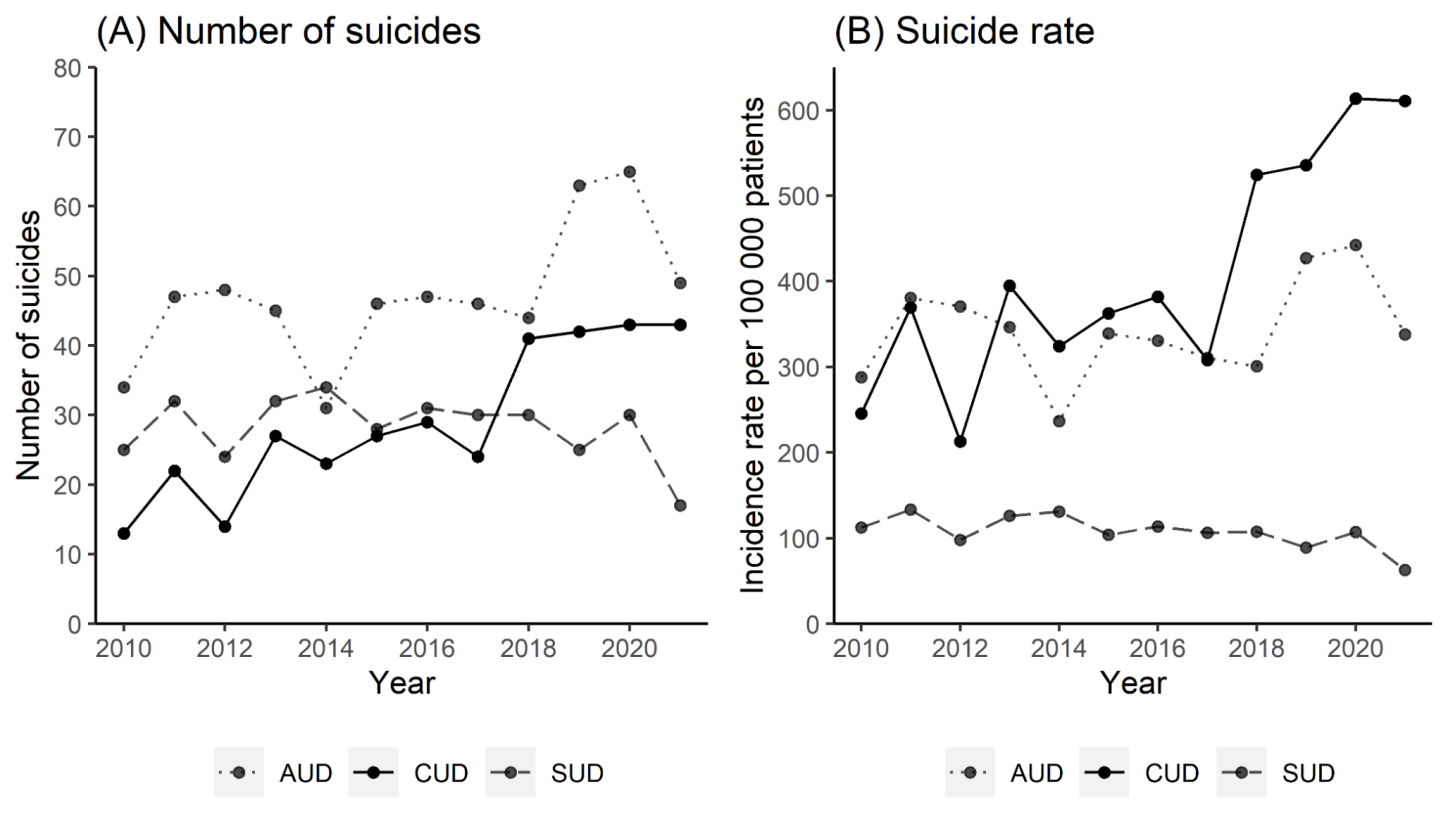
Annual number of suicides (panel **A**) and crude suicide rate (panel **B**) in patients with alcohol use disorder (AUD), cannabis use disorder (CUD) and other substance use disorder (SUD)

Patients with CUD were followed for 84,291 person-years and had an overall suicide rate of 412.9 per 100,000 person-years. Patients with AUD had an overall suicide rate of 343.8 per 100,000 person-years, and patients with other SUDs had an overall suicide rate of 107.1 per 100,000 person-years. Table 2 contains gender and age-stratified suicide rates. The annual number of suicides and suicide rates in patients with CUD had a slightly increasing trend from 2010 to 2017 before an increase was found from 2017 to 2018 and onwards (Fig. 1). The number of suicides increased from an annual mean of 22.4 (*SD* = 5.95) between 2010 and 2017, to 42.2 in the period between 2018 and 2021, where the number stabilized with less variation (*SD* = 0.95) (Fig. 1, panel A). The suicide rate followed a similar pattern and increased from a mean of 325 (*SD* = 66.3) between 2010 and 2017 to 571 (*SD* = 47.7) between 2018 and 2021 (Fig. 1, panel B). The suicide rate in patients with CUD showed an additional minor increase in 2020. Different trends were found for patients with AUD and other SUDs, though the suicide rate in patients with AUD also displayed an increase in 2019 and 2020 before returning to the previous level.

**Table 2:** Total, gender- and age-specific suicide mortality rates per 100,000 patients in the whole observation period 2010-21

Group		Cannabis use disorder			Alcohol use disorder			Other substance use disorders		
		Suicides	Patients	Rate	Suicides	Patients	Rate	Suicides	Patients	Rate
		*n*	*n*		*n*	*n*		*n*	*n*	
Total		348	84,291	412.9	565	164,325	343.8	338	315,525	107.1
Gender										
	Females	78	20,887	373.4	186	53,583	347.1	141	100,822	139.9
	Males	270	63,404	425.8	379	110,742	342.2	197	214,703	91.8
Age										
	10–19	11	4,967	221.5	< 3	1,758	113.8	4	4,938	81.0
	20–29	142	41,073	345.7	73	25,793	283.0	62	79,41	78.1
	30–39	117	22,693	515.6	93	30,885	301.1	94	96,265	97.6
	40–49	52	10,696	486.2	160	38,767	412.7	74	77,386	95.6
	50–59	15	4,214	356.0	136	40,638	334.7	66	43,645	151.2
	60-	11	648	1697.5	101	26,484	381.4	38	13,881	273.8

**Fig. 2:**
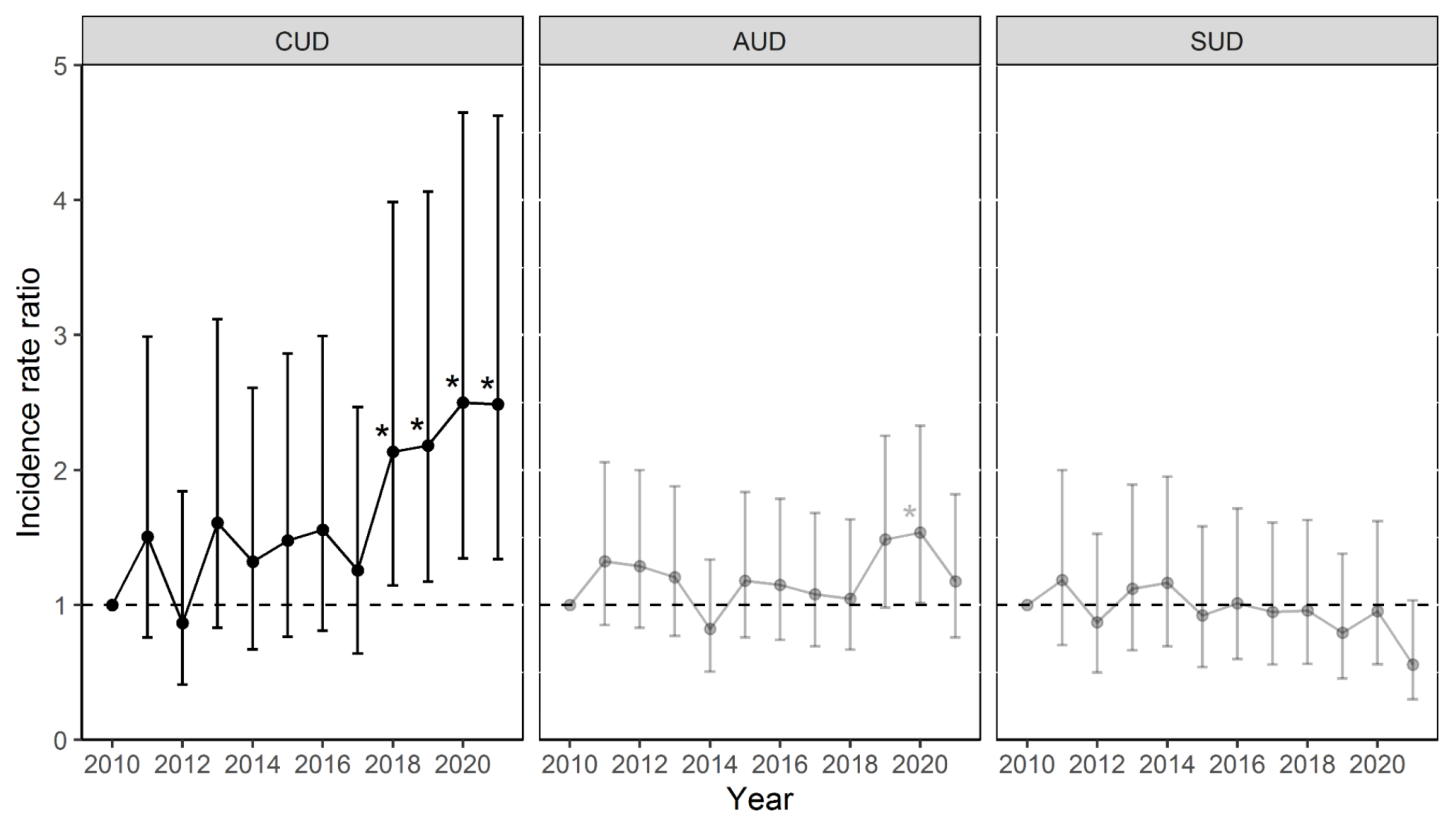
Annual incidence rate ratios compared to 2010 with corresponding confidence intervals in patients with alcohol use disorder (AUD) and cannabis use disorder (CUD). Significant incidence rate ratios are illustrated with asterisks in the plot

Compared with the year 2010, IRRs were significantly increased for patients with CUD in 2018 (IRR = 2.14 (95% CI 1.14–3.99), *p* = 0.017), 2019 (IRR = 2.18 (1.17–4.06), *p* = 0.014), 2020 (IRR = 2.50 (1.34–4.65), *p* = 0.004), and 2021 (IRR = 2.48 (1.34–4.62), *p* = 0.004). Annual IRRs are illustrated in Fig. 2. For patients with AUD, an increased IRR was found in 2019 compared to 2010 (IRR = 1.54 (1.02–2.33), *p* = 0.042). For other SUDs, no significant differences were found in the annual IRRs.

The time trend of the suicide rate in patients with CUD, modeled through Poisson regression (Table 3), was significant in the crude model in CUD patients (IRR = 1.08 (1.05–1.12), *p* = < 0.001), with an 8% annual increase in the suicide rates observed. Gender and age adjustment in Model B provided only minor changes in the estimate (IRR = 1.07 (1.04–1.11), *p* = < 0.001). In the crude or gender and age-adjusted models no significant trends were observed for patients with AUD or other SUDs (Supplementary tab. S1). Given the lack of a crude association between year and suicide rates in patients with AUD or other SUDs, covariates were not further considered for these groups. Adjustment for depressive or anxiety disorders attenuated the association between the suicide rate and the year (IRR = 1.00 (0.92–1.08), *p* = 0.999), while the covariate depressive or anxiety disorders was significant (IRR = 1.03 (1.00-1.06), *p* = 0.037). When adjusting for AUD or other SUDs attenuation was also observed (IRR = 0.96 (0.87–1.06), *p* = 0.408) with a significant covariate (IRR = 1.05 (1.01–1.09), *p* = 0.009). These findings suggests that depression or anxiety disorders, or alcohol or other SUDs may explain the increasing trend in the suicide rate in patients with CUD. No such association was found for psychosis or bipolar disorders, personality disorders, or ADHD in patients with CUD.

**Table 3:** Time trend in suicide rate in patients with cannabis use disorders regressed over years using Poisson regression

	Year		Covariate	
Model	IRR (95% CI)	*p*	IRR (95% CI)	*p*
Crude	1.08 (1.05–1.12)	**< 0.001**		
Gender and age-adjusted	1.07 (1.04–1.11)	**< 0.001**		
Adjusted for non-affective psychosis or bipolar disorder	1.06 (1.00-1.12)	**0.046**	1.02 (0.98–1.06)	0.362
Adjusted for depression or anxiety disorders	1.00 (0.92–1.08)	0.999	1.03 (1.00-1.06)	**0.037**
Adjusted for personality disorders	1.08 (0.99–1.16)	0.063	1.00 (0.96–1.06)	0.832
Adjusted for ADHD	1.07 (1.01–1.12)	**0.012**	1.01 (0.98–1.05)	0.427
Adjusted for alcohol or other substance use disorders	0.96 (0.87–1.06)	0.408	1.05 (1.01–1.09)	**0.009**

## Discussion

In this study, we found that the suicide rate doubled in patients with CUD from 2010 to 2021. Overall, an 8% yearly increase in suicide rates was found in CUD patients in the study period. Support was found for a concurrent increase in comorbid depressive or anxiety disorders and alcohol or other SUDs in these patients, which may explain some of the association during the period. No similar trend of changes in suicide rates throughout the period was observed for patients with AUD or other SUDs. Moreover, patients with CUD in general and patients with CUD who died by suicide were more often male and considerably younger than patients with AUD or other SUDs.

While there was a slight, non-significant increase in the suicide rate of CUD patients from 2010 to 2017, a substantial increase was observed in 2018 and 2019 before an increase of a smaller magnitude occurred in 2020 and 2021. This could result from changes in the services due to the COVID-19 pandemic, where restrictions and lockdowns started in Norway in March 2020. However, no overall change in the suicide rates in the patient population has been found during the COVID-19 pandemic [29], and it seems unlikely that the pandemic should affect suicide rates in CUD patients and not in AUD and other SUDs. Also, this does not explain the large increase from 2017 to 2018. During the study period, the suicide rate in the general Norwegian population was stable [30].

Adjusting the overall trend for depressive or anxiety disorders or other SUDs attenuated the overall association, pointing to two factors that could partly explain the increase in annual suicide rates. First, since depressive or anxiety disorders are strong risk factors for suicide [21], this seems plausible. We do not, however, know to what extent the mental disorders were caused by cannabis use, and hence, can be viewed as mediators in the association between CUD and suicide risk and to what extent they were present before the cannabis use onset. The second explanation examined was concurrent increases in AUD or other SUDs, which could point to a potential risk associated with either severity, comorbid substance use, or transitions from CUD into other SUDs. Further research is necessary to better understand the role of the potential explanatory factors we have identified.

A possible contributing explanation could be the increase in THC concentration in cannabis products seen during the recent years [31]. Higher cannabis potency is associated with an increased risk of adverse health outcomes such as dependence and mental disorders, including psychosis [32, 33]. As this study did not investigate the association between THC concentration and suicide directly, the possible contributing effect of stronger cannabis products can only be indirectly inferred based on coinciding trends. Such trends are also seen in an increase in treatment-seeking behavior for CUD observed in Europe [11] and an increase in the annual incidence of cannabis-induced psychosis in all Scandinavian countries [13]. This speculation is strengthened by the fact that we did not observe a similar increase in suicide rates in people with AUD and other SUDs, suggesting that possible explanations should be related to cannabis.

A significantly longer time from the incident of receiving any SUD diagnosis to suicide was observed for CUD relative to AUD and other SUDs. This increased time may suggest that a longer pathogenesis for suicidal behavior is more common in patients with CUD, compared to suicide risk in other SUDs; this corresponds well with previous findings that there is no acute effect of cannabis on suicidal behavior or suicide death [17].

Patients with CUD who died by suicide differed from those with AUD and other SUDs in several ways. They were more often male and younger, used hanging or strangulation as a method of suicide, and were first diagnosed in SUD services. In general, men more often use methods with higher case fatality rates, such as hanging or strangulation, than women [34]. However, adjusting for gender did not substantially alter the results. Furthermore, the young age of CUD suicides reflects the younger age of patients with CUD. Similar increases in risk for the male gender and younger ages have also been reported previously for the risk of developing CUD [17] and the risk of developing schizophrenia among individuals with CUD [35]. However, as almost three-fourths of suicide cases with CUD were aged below 39 at the time of their death, this results in a comparatively higher number of years lost incurred by CUD. This disproportionate harm of suicide in young CUD patients may be an important factor to consider regarding the prevention of suicide death in this group.

### Limitations

We only had individual-level data for those patients who died in suicide. In the total patient group, we only had aggregated data. This limited our ability to examine individual-level associations. This may particularly affect the adjustment for comorbid mental disorders where individual-level associations may be relevant. Furthermore, it limited the analytic possibilities to adjust models further or to employ other statistical models. Moreover, Norway has a small population, which, combined with a rare event at the population level, results in a limited number of annual cases, which limits our possibilities to examine subgroups and provide adjusted rates with adequate confidence intervals.

The patients who died by suicide were included in this study based on their contact with specialized mental health or SUD services within the last year. Patients with contacts before the previous year but with no contact during the last year were not included. Our sample is thus restricted to a high-risk population with recent contact with services. This may have affected our estimates, and the generalization of our findings should be limited to clinical populations.

The coding of the cause of death by suicide is strict in Norway. A lethal intoxication that is not accompanied by clear signals of intention to die, such as a suicide note, will be coded as intoxication, not suicide. This may partly explain the relatively low number of suicides in the group with other SUDs, which includes many individuals with opioid use disorder who are in opioid agonist treatment. Patients initiating and stabilized at such treatment may be less at risk of suicide [36]. Additionally, if they die by intoxication, it is less likely to be coded as suicide.

## Conclusions

We found increasing suicide rates in patients with CUD treated in specialized health services in Norway from 2010 to 2021. The increase was specific for CUD and not present in patients with AUD and other SUDs. This finding could be caused by more potent cannabis being on the market. Treatment providers need to be aware of this change in suicide rates. This illustrates the need to monitor suicide rates in the context of changes in consumption patterns or substances; such data are important to adapt preventive policies to fit the landscape. More knowledge about factors that affect suicide risk in patients with CUD is needed.

## Electronic supplementary material

Below is the link to the electronic supplementary material.

Supplementary Material 1

## Data Availability

Data is not publicly available due to confidentiality restrictions.
