# Supplementary material for: The annual trend of suicide rates from 2010 to 2021 in patients with cannabis use disorder – a national registry study
Source: Soc Psychiatry Psychiatr Epidemiol. 2024 Oct 28;60(7):1603–11. doi: 10.1007/s00127-024-02781-4 (PMC12238203; doi:10.1007/s00127-024-02781-4)
Supplement: Supplementary file 1 — Supplementary Material 1 [file 127_2024_2781_MOESM1_ESM.docx]

**Supp 1**. Time trend in suicide rate in patients with alcohol and other substance use disorders regressed over years using Poisson regression.

|  |  | Year | |  | Covariate | |
| --- | --- | --- | --- | --- | --- | --- |
| Model | | IRR (95 % CI) | *p* |  | IRR (95 % CI) | *p* |
| Alcohol use disorder | |  |  |  |  |  |
|  | Crude | 1.02 (0.99-1.04) | 0.201 |  |  |  |
|  | Gender and age-adjusted | 1.01 (0.99-1.04) | 0.163 |  |  |  |
|  | Adjusted for non-affective psychosis or bipolar disorder | 1.01 (0.98-1.03) | 0.516 |  | 1.03 (1.01-1.06) | **0.012** |
|  | Adjusted for depression or anxiety disorders | 0.97 (0.93-1.01) | 0.099 |  | 1.02 (1.01-1.04) | **0.002** |
|  | Adjusted for personality disorders | 0.99 (0.96-1.02) | 0.600 |  | 1.03 (1.01-1.04) | **0.010** |
|  | Adjusted for ADHD | 0.98 (0.95-1.02) | 0.385 |  | 1.06 (1.01-1.12) | **0.011** |
| Other substance use disorders | |  |  |  |  |  |
|  | Crude | 0.97 (0.93-1.00) | **0.037** |  |  |  |
|  | Gender and age-adjusted | 0.96 (0.93-0.99) | **0.005** |  |  |  |
|  | Adjusted for non-affective psychosis or bipolar disorder | 0.96 (0.93-0.99) | **0.019** |  | 1.02 (0.98-1.06) | 0.260 |
|  | Adjusted for depression or anxiety disorders | 0.95 (0.92-0.99) | **0.009** |  | 1.05 (1.00-1.09) | **0.035** |
|  | Adjusted for personality disorders | 0.97 (0.94-1.00) | 0.054 |  | 1.01 (0.98-1.05) | 0.421 |
|  | Adjusted for ADHD | 0.94 (0.90-0.98) | **0.010** |  | 1.09 (0.98-1.20) | 0.105 |
